# The landscape of fear as an emergent property of heterogeneity: Contrasting patterns of predation risk in grassland ecosystems

**DOI:** 10.1002/ece3.3021

**Published:** 2017-05-24

**Authors:** Fidelis Akunke Atuo, Timothy John O'Connell

**Affiliations:** ^1^ Department of Natural Resource Ecology and ManagementOklahoma State UniversityStillwaterOKUSA

**Keywords:** avian predators, habitat complexity, landscape of fear, Northern Bobwhite, predation risk, vegetation structure

## Abstract

The likelihood of encountering a predator influences prey behavior and spatial distribution such that non‐consumptive effects can outweigh the influence of direct predation. Prey species are thought to filter information on perceived predator encounter rates in physical landscapes into a landscape of fear defined by spatially explicit heterogeneity in predation risk. The presence of multiple predators using different hunting strategies further complicates navigation through a landscape of fear and potentially exposes prey to greater risk of predation. The juxtaposition of land cover types likely influences overlap in occurrence of different predators, suggesting that attributes of a landscape of fear result from complexity in the physical landscape. Woody encroachment in grasslands furnishes an example of increasing complexity with the potential to influence predator distributions. We examined the role of vegetation structure on the distribution of two avian predators, Red‐tailed Hawk (*Buteo jamaicensis*) and Northern Harrier (*Circus cyaneus*), and the vulnerability of a frequent prey species of those predators, Northern Bobwhite (*Colinus virginianus*). We mapped occurrences of the raptors and kill locations of Northern Bobwhite to examine spatial vulnerability patterns in relation to landscape complexity. We use an offset model to examine spatially explicit habitat use patterns of these predators in the Southern Great Plains of the United States, and monitored vulnerability patterns of their prey species based on kill locations collected during radio telemetry monitoring. Both predator density and predation‐specific mortality of Northern Bobwhite increased with vegetation complexity generated by fine‐scale interspersion of grassland and woodland. Predation pressure was lower in more homogeneous landscapes where overlap of the two predators was less frequent. Predator overlap created areas of high risk for Northern Bobwhite amounting to 32% of the land area where landscape complexity was high and 7% where complexity was lower. Our study emphasizes the need to evaluate the role of landscape structure on predation dynamics and reveals another threat from woody encroachment in grasslands.

## 1 Introduction

An animal's use of space within its home range is in large measure determined by competing pressures to acquire food, mates, or other needs while avoiding predation. In addition to the various vegetation and land cover patches that comprise the physical landscape of a home range, many animals perceive and respond to a landscape of fear defined by spatially heterogeneous risk of predation (Laundré et al., 2014). In the seminal study illustrating the concept, Laundré, Hernández, and Altendorf (2001) described increases in predator vigilance and corresponding decreases in foraging time among some elk (*Cervus elephas*) exposed to new threats of predation from reintroduced wolves (*Canis lupus*) following a 50‐year absence in Yellowstone National Park, USA. In addition to expanding to different species of predators and prey, subsequent work has addressed both temporal and spatial variability in the landscape of fear (Tolon et al., 2009) and experimental approaches to better quantify lost foraging time due to the perceived threat of predation (Matassa & Trussell, 2011). These studies suggest both that the landscape of fear has merit as an organizing theory in ecology and that the non‐consumptive effects of predators can have greater influence on the spatial use and prey demography than direct loss to predation (Cresswell, 2008; Laundré et al., 2014; Luttbeg & Kerby, 2005; Matassa & Trussell, 2011). Landscapes of fear are dynamic according to changes in predator populations and, presumably, changes in land cover that affect the spatial distribution of predators.

Complex vegetation structures are known to mediate predator–prey interactions by influencing predator's ability to search for, encounter, kill, and consume prey items (Gorini et al., 2012) or by providing refugia that allows prey species to escape predation. There is also evidence that structural complexity often increases the abundance and diversity of generalist predators with attendant consequences for prey species (Gorini et al., 2012; LaManna, Hemenway, Boccadori, & Martin, 2015; Oliver, Luque‐Larena, & Lambin, 2009). Conversely, when predators are suppressed or excluded from structurally complex landscapes, prey gains refugia from predation often resulting in increased population (Denno, Finke, & Langellotto, 2005). Temperate grasslands are dynamic ecosystems that in recent decades have experienced widespread increases in woody vegetation in many ecoregions. In addition to expansion of industrial agriculture, changes in climate, CO_2_ concentration, livestock grazing, and fire frequency have been specifically implicated as drivers (Coppedge, Fuhlendorf, Harrell, & Engle, 2008; Fuhlendorf, Archer, Smeins, Engle, & Taylor, 2007; Twidwell, Fuhlendorf, Taylor, & Rogers, 2013). In the Southern Great Plains of the United States, woody encroachment in natural grasslands is likely to introduce structural complexity that benefits avian predators and increases the vulnerability of their prey (Preston, 1980). For predators that typically hunt from perches, the presence or increase of woody cover in grasslands expands their effective hunting radius, thus increasing the “vulnerability landscape” for their prey species. Complex vegetation structure has been shown to increase the abundance and diversity of generalist predators with attendant consequences for prey (Gorini et al., 2012; LaManna et al., 2015; Oliver et al., 2009). For example, Andersson, Wallander, and Isaksson (2009) found that predator hunting efficiency increased with perch availability and perch height in open landscapes. Compared to hovering, predators hunting from perches minimize their energy cost and are able to increase their prey detection and capture efficiency (Leyhe & Ritchison, 2004; Tomee, Dias, Chumbinho, & Bloise, 2011).

Changes in land cover of grasslands have contributed to long‐term population declines in grassland birds (Brennan & Kuvlesky, 2005; Peterjohn & Sauer, 1999). Population declines have been attributed to several factors including land use change due to habitat fragmentation, pesticide use, and predation (Askins, 1993; Bogard & Davis, 2014; Mineau & Whiteside, 2013; Vickery, Herkert, Knopf, Ruth, & Keller, 1995). Upland game birds have particularly been affected by these changes with population trends of several species plummeting to historical lows (Aldridge et al., 2008; Hagen, Sandercock, Pitman, Robel, & Applegate, 2009; Hernández, Brennan, DeMaso, Sands, & Wester, 2013). The Northern Bobwhite (*Colinus virginianus*), for example, has experienced the most severe long‐term population decline of any North American bird species (Sauer et al., 2011) and is listed as “Near Threatened” under the IUCN/BirdLife threat criteria (BirdLife International 2016). Since 2000, the United States’ population is estimated to be declining at 3.7% annually (Sauer et al., 2011). Predators can play a large role independently or compensatory to expedite such declines (Sinclair et al., 1998). For example, in the Langholm Moor of southern Scotland, Thirgood, Redpath, Rothery, and Aebischer (2000) demonstrated that raptor predation alone accounted for 70% of winter and 90% of summer predation mortality in Red Grouse (*Lagopus lagopus scotica*). Two main predators, Peregrine Falcon (*Falco peregrinus*) and Hen Harrier (*Circus cyaneus*, conspecific with Northern Harrier), were responsible for all predation incidences in this species. Although the influence of direct predation pressure on prey population is often density dependent, often the mere presence of predators in the landscape can exert profound influence on prey behavior and vulnerability in a density non‐dependent way (Cresswell, 2008).

The role of spatial heterogeneity in mediating predator–prey interactions has been established in some experimental studies (Banks & Gagic, 2016; Chalfoun & Martin, 2009; Pacala, Hassell, & May, 1990), but replication in natural landscapes has received less attention. Where studied in the field, the importance of spatial heterogeneity in enhancing predators’ ability to kill prey (Lecomte, Careau, Gauthier, & Giroux, 2008; Oliver et al., 2009; Zub, Sönnichsen, & Szafrańska, 2008) or prey's ability to evade predators (Kauffman et al., 2007; Warfe & Barmuta, 2004) has been confirmed. However, the degree to which heterogeneity influences both predators and prey suggests that the perceived landscape of fear could be an emergent property of the physical landscape that variably increases and decreases the likelihood of overlap between two or more species of predators.

**Figure 1:**
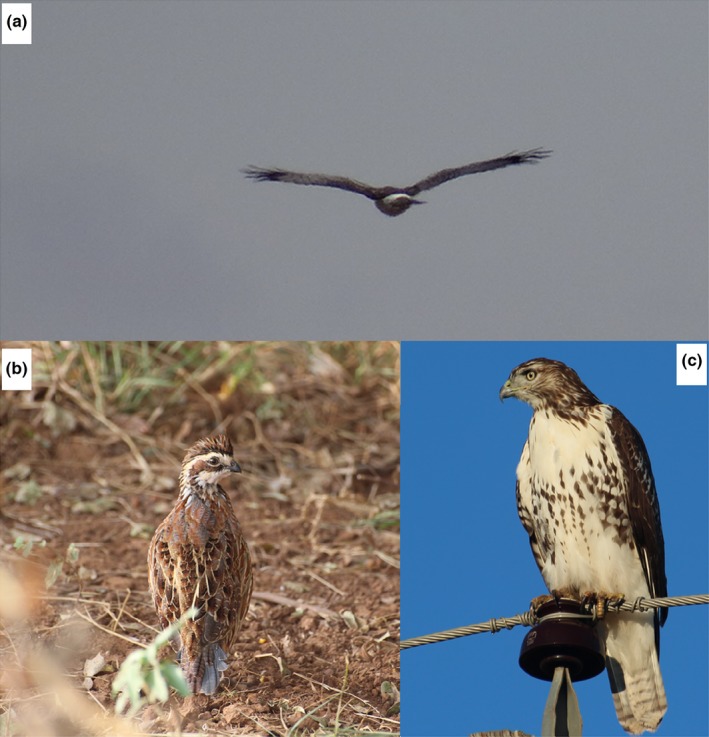
Photos of (a) Norther Harrier hovering over the prairie, (b) Northern Bobwhite, and (c) perched Red‐tailed Hawk

The mixed‐grass prairie of western Oklahoma provides unique opportunities for investigating the role of landscape complexity in altering predation risk for declining prey species. The region is dominated by mixed grasses, but woody encroachment in recent decades has dramatically altered landscape structure (Hall, 2015). The region supports multiple species of migratory diurnal raptors as either overwintering residents or stopover migrants. Many of these predators rely on similar sources of food; hence, increased diversity and abundance during these periods could constitute additive threats to vulnerable declining prey species such as Northern Bobwhite (Figure 1).

In the Southern Great Plains, Northern Harrier (Figure 1) and Red‐tailed Hawk (*Buteo jamaicensis*; Figure 1) are the most frequent avian predators of Northern Bobwhite (Turner et al., 2014). In this study, we aimed to: (1) examine the role of structural complexities on the fine‐scale distribution of two avian predators; (2) evaluate predation risk for quail across a gradient of vegetation complexity; and (3) map the overlap between predator habitat selection and Northern Bobwhite vulnerability to quantify a landscape of fear in physical landscapes that vary in heterogeneity.

## 2 Materials and Methods

### 2.1 Study sites

We conducted our study at Packsaddle and Beaver River Wildlife Management Areas (WMAs) in northwestern Oklahoma. Packsaddle WMA covers ~6,475 ha of mixed‐grassed prairie with an elevation that ranges approximately 579–762 m above sea level. The average annual precipitation is ~53 cm, with the majority occurring during spring and summer (DeMaso, Peoples, Cox, & Parry, 1997). Detailed vegetation and landscape characteristics of the area are described in DeMaso et al., 1997; Hall, 2015;. Beaver River WMA is ~7,163 ha in area, consisting of a mixture of upland, floodplain, and river bottom with a mean annual precipitation of ~7.6 cm. Vegetation around the upland area is predominantly sagebrush (*Artemsia filifolia*) and buffalograss (*Bouteloua dactyloides*) interspersed with sand plum (*Prunus angustifolia*) thickets and gently rolling sandhills. The floodplain portion of the WMA is comprised mostly of grasses mixed with cottonwood (*Populus deltoides*), hackberry (*Celtis occidentalis*), and American elm (*Ulmus americana*). The river bottom is made of mostly woody vegetation consisting of sand plum thickets and salt cedar (*Tamarix* spp) (Tanner et al., 2015). The Oklahoma Department of Wildlife Conservation manages the two WMAs mainly for hunting and cattle grazing. Most of the management practices on the WMAs are intended to increase the population of the Northern Bobwhite and other game species. A combination of prescribed grazing by cattle and prescribed burns is conducted (mostly at Packsaddle) to produce and promote the growth of native forbs. There is also significant oil exploration and extraction at Packsaddle WMA.

### 2.2 Raptor surveys

We collected raptor abundance data from 14 line transects at Packsaddle WMA and 16 at Beaver River WMA. Transects measured 2–5 km in length and were placed along existing roads based on protocols described in Fuller and Mosher (1987) and Bibby, Burgess, Hill, and Mustoe (2000). We surveyed each transect at least twice each month January 2013–December 2015. We placed transect lines non‐randomly along trails separated by a distance ≥900 m to reduce the chance of counting an individual more than once (Buckland et al., 2001). Surveys were carried out by one primary observer occasionally supported by a second person who acted primarily as a driver. The observer scanned a distance of approximately 400 m on either side of the transect line for raptors from a truck that was driven at a speed of 20–30 km/h (Andersen, 2007). For each detection, we estimated the distance of the bird from the transect line using a Nikon 8398 ACULON laser range finder. We georeferenced each detection at the point of observation using a Garmin Montana 650TM GPS unit. We also obtained the angle of observation from the observer using an azimuth compass. To develop a spatially explicit model of raptor habitat association, we plotted each georeferenced point to the point of actual occurrence in time using the “Bearing Distance to Line” tool in ArcGIS 10.2. This tool created a new feature class containing a geodetic line feature for each predator occurrence point constructed based on the values in the x‐ and y‐coordinate fields, angle of observation, and detection distance. Next, we used the “Feature Vertices to Points” tool to create a feature class containing a georeferenced points at the end of each geodetic line feature. These new points containing x‐, y‐coordinates represented the approximate location of individual birds at the time of detection. Thus, instead of merely recording the observation point from transects, we could plot offset points to more accurately reflect snapshots of spatially explicit occurrence.

### 2.3 Northern Bobwhite mortality sites

We obtained data on quail kill locations from collaborative and concurrent quail telemetry studies at Packsaddle and Beaver River WMAs. All kill sites were discovered through telemetry tracking of tagged birds. Trapping and tracking of quail occurred all through the year starting from spring of 2012 and ending in the autumn of 2015. Detailed descriptions of quail trapping and tracking techniques are provided in Carroll, Davis, Elmore, Fuhlendorf, & Thacker, 2015 for Packsaddle WMA and Tanner et al., 2015 for Beaver River WMA. Based on expert knowledge, Northern Bobwhite found dead were categorized into four mortality causes: (1) raptor predation, (2) mammal predation, (3) unknown predation, (4) non‐predation‐related mortality. We included only raptor‐specific predation events in our analysis. In all, we accumulated 179 raptor‐related quail mortality events at Beaver River and 210 at Packsaddle.

### 2.4 Habitat delineation

We quantified land cover used by Northern Harrier and Red‐tailed Hawk, and that surrounding kill sites for Northern Bobwhite, with 10‐m resolution imagery acquired through the National Agriculture Imagery Program (NAIP). The imagery was acquired in 2015 and had been pre‐processed and classified for the state of Oklahoma by the Oklahoma Department of Wildlife Conservation (ODWC) (http://www.wildlifedepartment.com/facts_maps/ecoregions.htm). The original land cover map contained 31 land cover classes for Packsaddle WMA and 24 classes for Beaver River WMA. We reclassified the land cover layer into nine dominant cover types at Packsaddle WMA (mixed grass, riparian woodland, riparian shrub, upland woodland, oil pads, upland shrub, open water, barren, and sandhill shinnery oak) and eight at Beaver River WMA (mixed grass, riparian woodland, riparian shrub, upland woodland, upland shrub, open water, pasture, and barren). In addition to vegetation attributes, we also calculated topographical attributes to include elevation, slope, and terrain roughness (degree of terrain ruggedness calculated as the standard deviation of elevation) from the digital elevation model (DEM) layer. We obtained DEM data from the United States Geological Survey (USGS) data portal at 1/3 arc‐seconds (10 m) resolution.

Based on the number of occurrence points (use locations) for Northern Harrier and Red‐tailed Hawk, we specified an equal number of random points to represent available locations using the random number generator in ArcGIS 10.2.2 (Environmental Systems Research Institute Inc., Redlands, CA, USA). To ensure that available points followed the same patterns as used points, we constrained random points within a 400‐m radius of each transect consistent with the detection distance. We then developed concentric buffers of 1,000 m radius centered on used and available points of each species to represent their approximate territories (Arroyo, Leckie, Amar, McCluskie, & Redpath, 2014; Janes, 1984; Stout, Temple, & Cary, 2006) and extracted the proportion of individual land cover types within each buffer. Similarly, we imposed buffers of 500 m radius centered on quail kill and random non‐kill locations and extracted vegetation variables. Although kill locations were identified with specific coordinates from transmitters, we established the broad buffer around those coordinates to accommodate possible differences between actual kill location and the location where a transmitter was found. Predators are known to move their prey *postmortem* (Kemper, Court, & Beck, 2013) such that the foraging site may be several meters away from where the prey's vulnerable location. A concurrent study on Lesser Prairie‐Chicken (*Tympanuchus pallidicinctus*) in western Oklahoma found that the average distance between last living location (last transmitter signal) and first mortality location was 546 ± 165 m (*n* = 21) based on a 1‐hr period (Ashly Unger, *personal communication*). In that same study, live prairie chickens move ~128 m in 1 hr, suggesting that the dead birds were moved *postmortem*.

### 2.5 Data analysis

We estimated average Northern Bobwhite seasonal vulnerability between the breeding and non‐breeding season by comparing monthly mortality rates of quail per hectare. Also, we estimated average mortality rate at each site based on the number of mortality events per hectare.

Given that quail vulnerability to raptor predation may differ across seasons, we examined temporal patterns in predator densities for the breeding and non‐breeding seasons of the Northern Bobwhite. We estimated detection‐corrected densities of Red‐tailed Hawk and Northern Harrier using program Distance 6.2 (Thomas et al., 2010). We estimated distance detection functions using the multiple‐covariate distance sampling (MCDS) approach (Buckland, Rexstad, Marques, & Oedekoven, 2015; Marques, Thomas, Fancy, Buckland, & Handel, 2007). The detection function model estimates detection probabilities with increasing distances from transect lines. For each study site, we compared a suite of a priori candidate models including half‐normal, hazard‐rate, and uniform function keys with cosine adjustment terms. We included different covariates (time of the day, month of survey, observer ID, and their interactions) to increase the explanatory power of our models. We then ranked models using Akaike's information criterion (AIC) and collected density estimates and detection probabilities based on the best competing models. Best models were those within a ΔAIC window of <2.

We used resource selection functions (RSFs) (Manly et al. 2002) to assess Red‐tailed Hawk and Northern Harrier habitat selection and Northern Bobwhite vulnerability. We used the generalized linear mixed model (GLMM) approach to compare environmental variables collected at raptor occurrence points to those collected from random points. We followed the same approach to compare environmental variables collected at Northern Bobwhite kill sites to those collected from randomly selected non‐kill locations. In both cases, we specified binomial error structures and included year as a random effect to account for variation in predator abundances or Northern Bobwhite mortality across three sampling seasons. For each model, we defined fixed effects to include vegetation, topographical, and distance (measured as Euclidean distance between used or available points and identified features in the landscape) variables. We performed a Pearson correlation on all variables to check for multicollinearity. In the absence of significant correlations (|*r*| > .7), we included all measured variables in our models.

To reduce complexity for all RSF models, we employed a two‐step approach to build habitat selection models. First, we evaluated all possible model combinations derived from the main effects of raptor habitat use, main effects of Northern Bobwhite vulnerability, and the interaction effects in both cases. In the second step, we selected the most important models from the all possible candidate model sets based on its AICc values (Burnham & Anderson, 2002). Using these models as bases, we evaluated the possibility of improving model fit by examining the additive and interactive roles of additional covariates (Züur, Ieno, Walker, Saveliev, & Smith, 2009). When the additional covariate improved the starting model (i.e., has a lower log‐likelihood), it was retained; otherwise, it was removed and the iterative process continued. We then ranked all candidate models according to their AIC values adjusted for small sample size (Burnham & Anderson, 2002) using the MuMIn package (Barton, 2016). We considered competing models within a ΔAICc < 2 as important in explaining habitat selection in raptors or landscape vulnerability in Northern Bobwhite. We evaluated model‐averaged estimates for variables of interest in competing models and calculated unconditional standard errors and 95% confidence limits (Burnham & Anderson, 2002). Following model averaging, we obtained *p*‐values based on top models within a ΔAICc < 2. Prior to statistical analysis, we standardized all environmental variables to a mean of 0 and a standard deviation of 1 to improve data interpretations. We used parameter estimates from our RSF models to generate maps of relative probability of use for Northern Harrier and Red‐tailed Hawk and relative predation risk for Northern Bobwhite. We created distance rater layers from distance covariates (i.e., distance to roads, distance to oil pads, and distance to river) and included individual layers of slope, elevations, and ruggedness to our model.

To compare the resulting output map of Northern Bobwhite predictive predation risk to those of Red‐tailed Hawk and Northern Harrier habitat use, we converted the continuous predictive values of each map to categorical outputs based on pre‐set cutoff thresholds. For each species, we specified a threshold of relative probability of use or vulnerability based on the methods described in York et al. (2011). Using the relative predictive maps produced for quail vulnerability and raptor habitat suitability, we extracted probability values for each map based on 100 random. For each map, we defined a threshold corresponding to the median probability value. We categorized all values above the threshold as used (raptors) or vulnerable (quail) and values below the threshold as avoided (raptors) or safe (quail) areas.

We then multiplied the three categorical raster layers in “raster calculator” to obtain a categorical map with eight classes, each representing the degree of Northern Bobwhite predation risk (1 = low use by Red‐tailed Hawk, low use by Northern Harrier, and low risk to Northern Bobwhite; 2 = low use by Red‐tailed Hawk, low use by Northern Harrier, but high risk to Northern Bobwhite; 3 = high use by Northern Harrier, but low risk to Northern Bobwhite; 4 = high use by Northern Harrier and high risk to Northern Bobwhite; 5 = high use by Red‐tailed Hawk but low risk to Northern Bobwhite; 6 = high use by Red‐tailed Hawk, but high risk to Northern Bobwhite; 7 = high use by Red‐tailed Hawk and Northern Harrier, but low risk to Northern Bobwhite; 8 = selected by Red‐tailed Hawk and Northern Harrier, and high risk to Northern Bobwhite). Then, we calculated the proportion of each predation risk category relative to our study area based on the number of pixels in each category.

Next, we compared levels of vegetation complexity (number of vegetation classes) across three important predation risk classes; i.e., areas where Northern Bobwhite was vulnerable to (1) Northern Harrier only, (2) Red‐tailed Hawk only, and (3) both Red‐tailed Hawk and Northern Harrier (double predator overlap). To do this, we generated 60 random points for each predation risk class. Next, we developed buffers of 100 m radius centered on each random point and extracted the number of pixels representing each vegetation cover type. We selected 100 m radius buffers in order to minimize the number of vegetation classes. We then compared vegetation complexity across the three predation risk classes using a two‐way analysis of variance (ANOVA) approach. We include an interaction term to examine the effects of site and vegetation use per predator type. We used Tukey's HDS test to assess within‐group variation. Except for density analysis, we performed all statistical analyses in program R version 3.3.1 (R Core Team 2015)

## 3 Results

**Figure 2:**
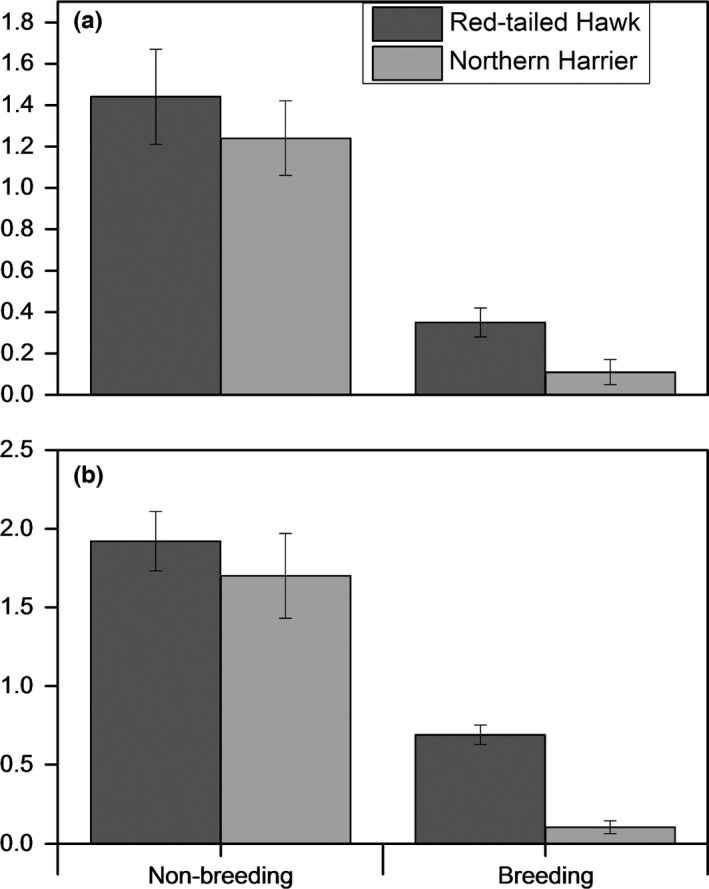
Mean densities of Northern Harrier and Red‐tailed Hawk during the breeding and non‐breeding seasons of the Northern Bobwhite at Packsaddle (a) and Beaver River (b) Wildlife Management Areas in western Oklahoma, USA, 2013–2015

Quail mortality rates were substantially high at Packsaddle with a monthly mortality rate of 0.61 per 100 ha. At Beaver River, monthly mortality rates were 0.32 per ha. At both sites, quail mortality rates were higher during the non‐breeding season (Packsaddle = .0.44 per 100 ha and Beaver River = 0.26 per 100 ha) compared to the breeding season (Packsaddle = .0.68 per 100 ha and Beaver River = 0.42 per 100 ha) suggesting a possible higher predation‐specific mortality for adult birds. Density of both Red‐tailed Hawk and Northern Harrier was higher during the non‐breeding season than during the breeding season (Figure 2). Compared to Northern Harrier, non‐breeding densities of Red‐tailed Hawk were slightly higher at each study site, but significantly (*p* < .05) higher during the breeding season (Figure 2).

We developed predictive maps depicting the relative landscape vulnerability of the Northern Bobwhite to avian predation risk at Packsaddle and Beaver River WMAs. The models for predictive mapping of quail vulnerability included vegetation composition, elevation, slope, distance to water, and distance to anthropogenic structures (e.g., oil pads). At Beaver River WMA, Northern Bobwhite appeared to be most vulnerable to raptor predation at higher elevations farther away from riparian woodland (Figure S1). Similarly, a vulnerability map for Packsaddle showed that Northern Bobwhites were more likely to be vulnerable to raptor predation at higher elevations, in mixed vegetation (mixture of shinnery oak shrub and grass cover) and in areas near oil pads (Figure S2). Predicted quail vulnerability was relatively low at low elevation close to riparian woodland. Overall, our results indicated a broader area of vulnerability at Packsaddle compared to Beaver River (Figures S2 and 3).

**Figure 3:**
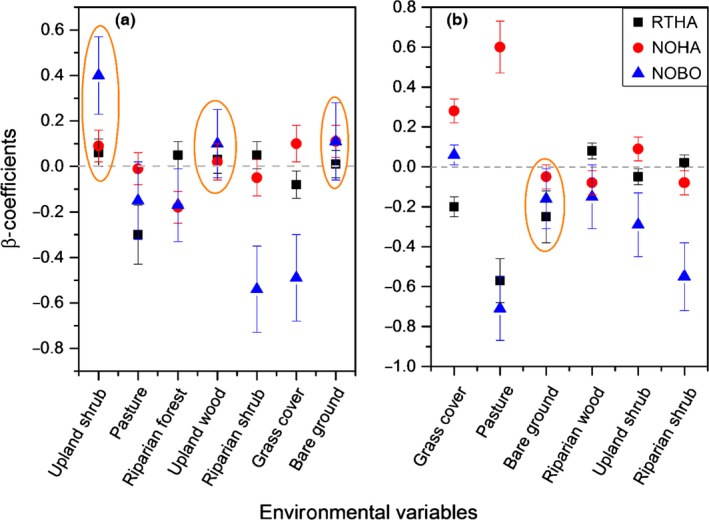
Beta coefficients of (β + 95% CI) for predicted habitat selection by Northern Harrier and Red‐tailed Hawk, and predicted Northern Bobwhite vulnerability, at Packsaddle (a) and Beaver River (b) Wildlife Management Areas in western Oklahoma, United States, 2013–2015. Ellipses indicates where overlaps of both predators resulted in significant quail mortality

We identified two competing models (ΔAICc < 2) to explain quail vulnerability at Packsaddle (Table S1a in Supporting Information). Both models included mixed grass, riparian shrub, upland woodland, and upland shrub. The two models were well supported receiving a cumulative Akaike weight of 0.61. We identified three competing models to explain Northern Bobwhite vulnerability at Beaver River (Table S1b). These models together accounted for 65% of the cumulative weight of evidence (AIC_*w*_ = 0.65) and included mixed grass, pasture, riparian shrub, riparian woodland, and upland woodland. Based on resource selection coefficients, the relative probability of Northern Bobwhite mortality at Packsaddle WMA was positively predicted by upland woody shrub, but negatively with increasing patches of mixed‐grass riparian shrub and riparian woodland (Figure 3a and Table S1b). At Beaver River, the likelihood of predation‐specific Northern Bobwhite mortality decreased significantly with grass and riparian woodland (Figure 3b and Table S1b).

The best supported models for predator resource selection at Packsaddle showed Red‐tailed Hawk selection positively associated with riparian woodland, upland shrub, upland woodland, and riparian shrub, but negatively associated with grass cover (Figure 3a). These variables were included in the top completive models (ΔAICc < 2) with a cumulative AICc weight of 0.73 (Table S2a). Conversely, Northern Harrier selection was positively associated with grass cover and upland shrub, but negatively with riparian woodland and riparian shrub cover. The top completing models identified grass cover and upland shrub as the most important variables for Northern Harrier selection in this landscape (Table S1b). At Beaver River, the most supported models for Red‐tailed Hawk selection included the variables riparian woodland, grass cover, and bare ground (Table S3). The likelihood of Red‐tailed Hawk selection was positively associated with riparian woodland cover, but negatively associated with grass cover and bare ground (Figure 3b). Northern Harrier selection was positively associated with grass cover, pasture, and upland shrub cover, but decreased with riparian woodland (Figure 3b). These variables were present in the top competing model (ΔAICc < 2) and received a cumulative AIC weight of 0.7 (Table S3).

### 3.1 Selection‐vulnerability overlap

**Figure 4:**
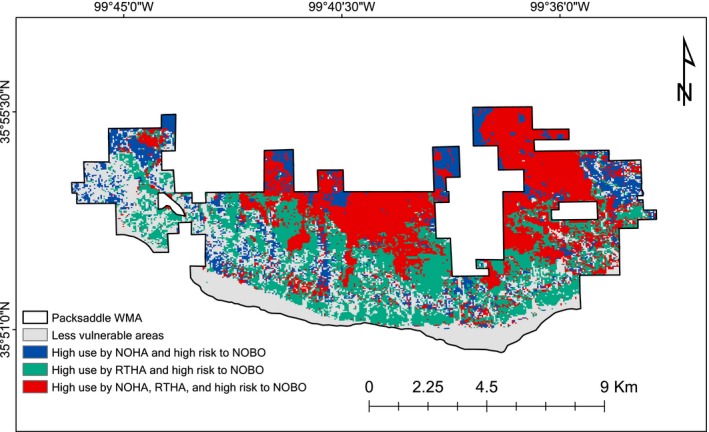
Predicted overlap map between Red‐tailed Hawk (RTHA) and Northern Harrier selection (NOHA), and Northern Bobwhite (NOBO) vulnerability at Beaver River WMA, Oklahoma, USA, 2013–2015

**Figure 5:**
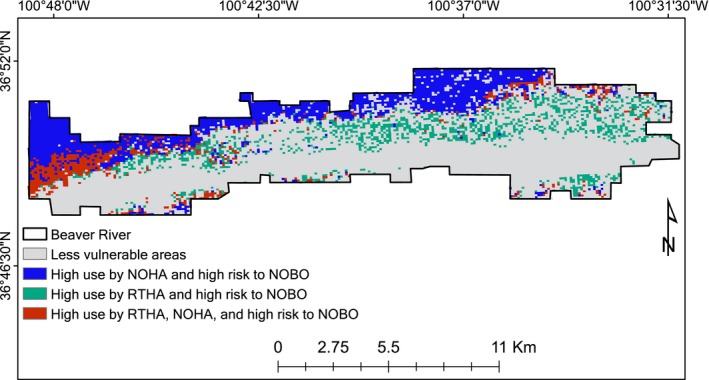
Predicted overlap map between Red‐tailed Hawk (RTHA) and Northern Harrier selection (NOHA), and Northern Bobwhite (NOBO) vulnerability at Packsaddle WMA, Oklahoma, USA, 2013–2015

We evaluated the degree of overlap between habitat selection of Red‐tailed Hawk and Northern Harrier and Northern Bobwhite vulnerability to identify areas of highest risk for Northern Bobwhite. We quantified habitat overlap into eight categories based on raster calculations. We considered areas where Northern Bobwhite vulnerability overlapped with both Red‐tailed Hawk and Northern Harrier selection (double predator overlap) as most risky areas for Northern Bobwhite. This high predation risk space represented ~32% of the total area of Packsaddle WMA (Figure 4) and only ~7% of Beaver River WMA (Figure 5) based on pre‐set selection‐vulnerability thresholds. Likewise, areas where Northern Bobwhite was least vulnerable to raptor predation constituted ~13% and ~33% for Packsaddle and Beaver River WMAs, respectively (Figures 4 and 5). At Packsaddle WMA, quail vulnerability overlapped more with Red‐tailed Hawk (~27%) than with Northern Harrier selection (~14%). This was different at Beaver River where the degree of overlap was higher with Northern Harrier (~24%) than with Red‐tailed Hawk (~6%).

**Figure 6:**
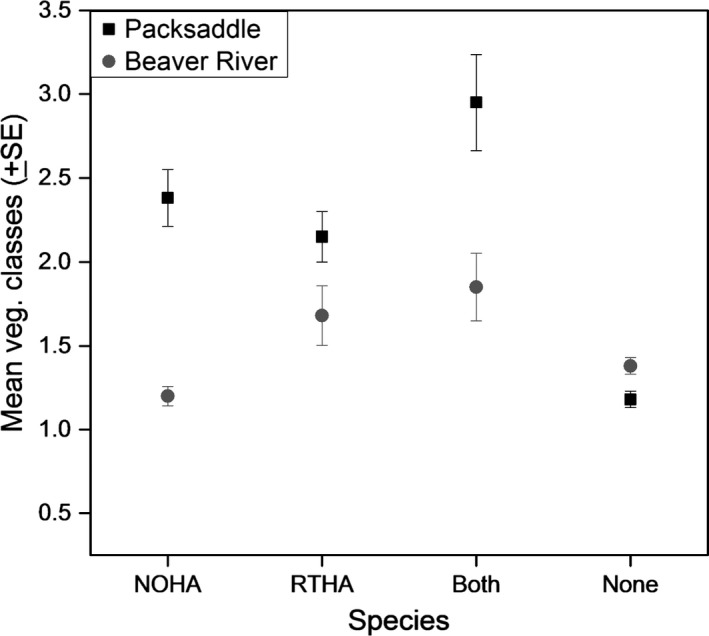
Mean levels of vegetation classes collected from 100 m radius plots of predicted Northern Bobwhite vulnerability to Northern Harrier (NOHA), Red‐tailed Hawk (RTHA), and Red‐tailed Hawk plus Northern Harrier (Both) at Packsaddle and Beaver River WMAs, Oklahoma, USA, 2013–2015

An examination of vegetation complexities across three contrasting areas of quail predation risk (i.e., vulnerable to Northern Harrier only, vulnerable to Red‐tailed Hawk only, and vulnerable to both Red‐tailed Hawk and Northern Harrier) revealed significant differences for both sites (*F*_7,539_ = 16.94, *p* < .001). Generally, we found greater vegetation complexities in areas of high vulnerability compared to least vulnerable areas (Figure 6). The interaction effect between site and vegetation use per predator type was also significant (*F*_3,539_ = 18.33, *p* < .001) indicating that quail vulnerability to different predator types due to vegetation complexity differs significantly by site.

At Packsaddle WMA, these differences were significantly greater (*p* < .001) for areas of double predator overlap than areas where quail vulnerability overlapped with individual predators (Figure 6). Similarly, vegetation complexity in areas of double overlap at Beaver River WMA was significantly higher compared to overlap with Northern Harrier but not Red‐tailed Hawk (post hoc test: *p* < .001; Figure 6).

## 4 Discussion

Our study assayed the potential predation risk of prey species in relation to top avian predators in mixed‐grass ecosystems with different heterogeneity gradients. Northern Bobwhite predation risk attributed to avian predators at both Packsaddle and Beaver WMA peaked in the winter. High quail mortality in the winter was consistent with the cumulative densities of Red‐tailed Hawk and Northern Harrier at that time of the year and was higher compared to the breeding season. Several species of raptors winter in the Southern Great Plains (Behney, Boal, Whitlaw, & Lucia, 2011, 2012). For example, Northern Harrier does not breed in any of our study sites but arrives as early as July and leaves late in spring. This migration pattern of the Northern Harrier in the Southern Great Plains was previously reported by Littlefield, Johnson, and Brush (2005). The arrival of these wintering visitors and the presence of overwintering residents create a robust and diverse suite of predatory birds that interact to increase predation pressure on vulnerable prey species. It is therefore plausible that the high vulnerability of Northern Bobwhite to predation at this time of the year is due to multiple attacks from predators with different hunting strategies. This creates a contrasting landscape of fear such that avoidance of one predator might increase predation risk to another, a phenomenon often referred to as “risk enhancement” (Sih, Englund, & Wooster, 1998). This has been well demonstrated in mammalian predator–prey interactions (Gorini et al., 2012). For example, a study examining predation risk to European Roe Deer (*Capreolus capreolus*) from humans and Eurasian Lynx (*Lynx lynx*) showed that the interaction of two predators created areas of contrasting risk that double predation risk for roe deer in the same landscape (Lone et al., 2014). Similarly, when facing with attack from two mammalian predators, elks’ avoidance of wolves by selecting denser vegetation cover exacerbated their risk of direct predation by cougars (Atwood, Gese, & Kunkel, 2009).

Low winter survival of Northern Bobwhite in the Southern Great Plains has been reported by previous studies and is largely attributed to predation and weather extremes (Cox, Peoples, DeMaso, Lusk, & Guthery, 2004; Holt, Burger, Leopold, & Godwin, 2012). Higher mortality of quail during winter translates to fewer individuals to spark population growth through reproduction during the subsequent spring and summer. Conservation efforts often focus on nest, chick, and juvenile survival (Schreiber et al., 2016; Trine, 1998), but a growing body of empirical evidence suggests that adult survival is sometimes critical population viability (Crouse, Crowder, & Caswell, 1987; Scheele et al., 2016; Weimerskirch, Brothers, & Jouventin, 1997). Resource selection analysis indicated clear differences in broad‐scale habitat use between Red‐tailed Hawk and Northern Harrier at the relatively homogeneous Beaver River WMA but greater overlap in the comparatively heterogeneous Packsaddle WMA. Red‐tailed Hawk employs a sit and watch strategy (Lish, 2015) that allows it to launch attack at its prey from a perch, while Northern Harrier forages by hovering low over grasslands, frequently changing its direction and pace in response to fine‐grained variation in habitat and prey availability (MacWhirter & Bildstein, 1996). This attribute allows the Northern Harrier to sight, pursue, and capture its prey (often ground dwelling birds, and rodents) with relative ease. The two species together constitute the most important avian predators of Northern Bobwhite in the Central and Southern Great Plains (Turner et al., 2014). At Beaver River, harriers showed a significant preference for uplands, selecting large patches of grassland vegetation, while Red‐tailed Hawks were primarily restricted to riparian woodlands. Both species however show great overlaps at Packsaddle, thus enlarging the landscape of predation risk for vulnerable prey species. We also found that spatial variation in Northern Bobwhite predation risk was a function of vegetation complexities inherent in a study system. At both study sites, quail were most vulnerable to predation at areas of high vegetation complexities. This was most evident in the relatively high mortality rates of Northern Bobwhite that we recorded at Packsaddle WMA. Compared to Packsaddle, vulnerable bobwhite habitats at Beaver River were localized with kill locations mostly concentrated in upland grassland, pastures, and upland shrub cover types.

Vegetation structure at Beaver River WMA provides large patches that allow avian predators to specialize. For example, upland vegetation at this landscape is predominantly grassland (Tanner et al., 2015) and mostly devoid of trees. This limits the hunting efficiency of “perch and hunt” predators such as Red‐tailed Hawk..Hence, prey species inhabiting these broad grassland patches are subjected to predation from the grassland specialist Northern Harrier, but they are generally immune to predation from Red‐tailed Hawk. At Packsaddle WMA, the vegetation is mixed shrub with mottes of hybrid shinnery oak distributed across the landscape (Hall, 2015). Shinnery oak (*Quercus havardii*) is typically <0.6 m tall, but those that have hybridized with postoak (*Quercus stellata*) often form mottes up to 6–8 m in height (Hall, 2015; Pettit, 1986). The distribution of these mottes in upland grasslands provides a network of elevated perches that creates conditions suitable for perch and hunt predators that normally avoid grassland (Behney et al., 2012). The availability of perches in open grassland at Packsaddle WMA increases the diversity of raptors in uplands, thus expanding the area of high predation risk across the landscape (Denno et al., 2005). Compared to Beaver River, Northern Bobwhite at Packsaddle may have to deal with predation risk from multiple avian predators. This elevated risk is often spatially correlated with a higher net mortality rate. In this case, the benefits from shifting habitats are small because avoidance of a predator in one vegetation cover type may result in exposure to another in a different cover type. A large body of evidence shows that when predation risk is homogeneous, it is easier for prey species to develop anti‐predation strategies that reduce net risk (Cresswell & Quinn, 2013; Sih et al., 1998; Thaker et al., 2011). Patterns of bobwhite mortality (Figures 4 and 5), together with low vulnerability risk at Beaver River, support this hypothesis. Furthermore, bobwhite vulnerability was widely spread across the Packsaddle landscape with significant kills occurring where woody vegetation mixed with grass cover. This is an indication that landscape‐level complexities resulting from reduction in patch sizes of unique vegetation types may create pockets of edge effects capable of reducing the amount of safe habitats for prey species (Denno et al., 2005). One anti‐predatory response of Northern Bobwhite to a modeled avian predator was to fly into dense vegetation of ~38 cm tall (Perkins, Boal, Rollins, & Perez, 2014). Therefore, alterations in the landscape matrix that changes the nature in which background refugia are nested, create an unstable dynamic in the ability of bobwhite to avoid their top predators. This creates a disparity between bird abundance and the proportion that avian predators can potentially kill. We saw a decrease in mortality as grassland patches increased in size. This is consistent with other studies on avian mortality resulting from raptor predation. Beyond a threshold distance of 30 m from predator cover, Redshank (*Tringa totanus*) predation by Eurasian Sparrowhawks (*Accipiter nisus*) decreased significantly and tended toward uniformity (Cresswell, Lind, & Quinn, 2010).

Despite their role in altering the predation risk gradient in grasslands ecosystems, mottes of hybrid shinnery oak act as important thermal refugia for ground dwelling birds during periods of high weather extremes. Carroll et al. (2015) noted that these tall shrubs reduced ground temperatures by 10°C more than other vegetation cover types during peak diurnal heating. Under extreme conditions, patches that might be avoided in a landscape of fear could be attractive in a “landscape of survival.” The predation–starvation risk hypothesis predicts that birds will avoid risky habitats until starvation risk exceeds predation risk (Cresswell & Whitfield, 2008). Yasué, Quinn, and Cresswell (2003) demonstrated that increased energy requirements from extreme cold were sufficient to increase use of conditionally beneficial patches normally avoided due to predation risk. Northern Bobwhite may trade off predation risk by seeking refuge in risky habitats during periods of extreme conditions. Such trade‐offs are seasonal, however, as predation risk from raptors is lower when shinnery oak mottes are sought for thermal refuge from high temperatures in summer.

Overall, we have identified a difference between two landscapes of varying complexity that corresponded to a difference in mortality rate of a focal species. Careful observation of habitat selection in two predators of that species revealed niche segregation in a more homogenous landscape of mixed prairie and herbaceous vegetation patches and broad niche overlap in a heterogeneous landscape of interspersed woody and herbaceous vegetation. We interpret the broad overlap of Red‐tailed Hawk and Northern Harrier in the heterogeneous landscape as an emergent property of land cover composition in that landscape that exposes potential prey species such as Northern Bobwhite to two predators that use different hunting strategies.

In grasslands in many parts of the world, land cover complexity is increasing through the invasion of woody vegetation. Although such changes can be subtle at first, grasslands can rapidly transit to landscapes of mixed herbaceous and woody cover. Should the woody cover increase in extent or stature to the point that it becomes an attractant for species previously not well supported in the grassland, it can render previously productive landscapes unproductive for species native to the grassland. This can occur through realized increases in mortality or nest loss or indirectly through avoidance of key resources now limiting through perceived risk of predation. Novel patches that occupy a small proportion of overall land cover have a disproportionate influence on landscape function. The good news for conservation is that where the expansion of a single land cover element can be associated with declining habitat quality for species of concern, potential solutions are possible with manipulation of those elements. In our system, for example, selective thinning or removal of a small number of hybrid shinnery oak patches at Packsaddle WMA could effectively remove interactions with Red‐tailed Hawk for Northern Bobwhites across large swathes of the landscape. Despite the generality of declining habitat quality for native species as novel land cover elements proliferate, specific management actions must rely on specific habitat use information of the species involved.

## Conflict of Interest

None declared.

## Author Contributions

F.A.A and T.J.O conceived the study. F.A.A and T.J.O designed methods. F.A.A collected and analyzed data. F.A.A. wrote the paper. Both authors contributed critically to the drafts and gave final approval for publication.
