# Supplementary material for: The landscape of fear as an emergent property of heterogeneity: Contrasting patterns of predation risk in grassland ecosystems
Source: Ecol Evol. 2017 May 24;7(13):4782–93. doi: 10.1002/ece3.3021 (PMC5496548; doi:10.1002/ece3.3021)
Supplement: Supplementary file 1 [file ECE3-7-4782-s001.docx]

**Online supporting information**

**Tables**

Table S1a: Model selection results of Northern Bobwhite vulnerability at Beaver River and Packsaddle Wildlife Management Areas in Oklahoma, USA, 2013 – 2015. For each study site, top 10 models are presented. Included for each model are number of parameters (K), Akaike’s Information Criterion values corrected for small sample sizes (AICc), delta AICc (ΔAICc), and Akaike weight (Ѡi).

| Site | Models | df | AICc | delta | w |
| --- | --- | --- | --- | --- | --- |
| Packsaddle | Grass+riparian shrub+upland wood | 4 | 390.86 | 0.00 | 0.38 |
|  | Grass+riparian shrub+riparian wood+upland shrub | 5 | 391.83 | 0.97 | 0.23 |
|  | Grass+riparian shrub+upland shrub+upland wood | 5 | 392.91 | 2.06 | 0.13 |
|  | Grass+riparian shrub+riparian shrub+riparian wood+upland wood | 6 | 393.90 | 3.04 | 0.08 |
|  | Grass+riparian shrub | 3 | 394.64 | 3.78 | 0.06 |
|  | grass+upland shrub | 3 | 395.65 | 4.79 | 0.03 |
|  | Grass+riparian shrub+riparian shrub | 4 | 396.40 | 5.54 | 0.02 |
|  | grass+upland shrub | 3 | 396.64 | 5.78 | 0.02 |
|  | Grass+riparian shrub+upland wood | 4 | 396.66 | 5.80 | 0.02 |
|  | Uplan shrub | 2 | 396.73 | 5.87 | 0.02 |
|  |  |  |  |  |  |
| Beaver | Grass+pasture+riparian shrub+upland wood | 5 | 462.40 | 0.00 | 0.30 |
|  | Grass+pasture+riparian shrub+riparian wood+upland wood | 6 | 463.37 | 0.97 | 0.18 |
|  | Grass+pasture+riparian shrub | 4 | 464.10 | 1.70 | 0.13 |
|  | Pasture+riparian wood+riparian shrub | 4 | 464.48 | 2.08 | 0.11 |
|  | Pasture+riparian shrub | 3 | 464.63 | 2.23 | 0.10 |
|  | Grass+pasture+riparian shrub+riparian wood | 5 | 465.37 | 2.96 | 0.07 |
|  | pasture+riparian shrub+riparian wood +upland shrub | 5 | 465.57 | 3.17 | 0.06 |
|  | pasture+riparian shrub+ riparian wood | 4 | 465.92 | 3.52 | 0.05 |
|  | Pasture+riparian wood | 3 | 472.79 | 10.39 | 0.00 |
|  | Grass+pasture+riparian wood | 4 | 472.91 | 10.51 | 0.00 |

Table S1b. Model-averaged coefficients estimating the predicted habitat selection for Red-tailed Hawk and Northern Harrier. Also included are model-averaged coefficients estimating the predicted habitat vulnerability for Northern Bobwhite (NOBO), at Packsaddle Wildlife Management Area in western Oklahoma, United States, 2013–2015.

|  | Red-tailed Hawk | | |  | Northern Harrier | | |  | NOBO mortality | | |
| --- | --- | --- | --- | --- | --- | --- | --- | --- | --- | --- | --- |
|  | B | SE | Pr(>\|z\|) |  | B | SE | Pr(>\|z\|) |  | B | SE | Pr(>\|z\|) |
| (Intercept) | -0.01 | 0.06 | 0.81 |  | -0.42 | 0.07 | < 0.01 |  | -0.65 | 0.07 | < 0.01 |
| Upland shrub | 0.06 | 0.06 | 0.29 |  | 0.09 | 0.07 | 0.18 |  | 0.40 | 0.17 | 0.02 |
| Pasture | -0.30 | 0.13 | 0.03 |  | -0.01 | 0.07 | 0.86 |  | -0.15 | 0.17 | 0.39 |
| Riparian forest | 0.03 | 0.06 | 0.55 |  | 0.18 | 0.07 | 0.01 |  | -0.17 | 0.16 | 0.31 |
| Upland wood | 0.02 | 0.06 | 0.66 |  | 0.00 | 0.08 | 0.98 |  | 0.00 | 0.15 | 0.98 |
| Riparian shrub | 0.01 | 0.06 | 0.86 |  | -0.05 | 0.08 | 0.52 |  | -0.54 | 0.19 | 0.01 |
| Grass cover | -0.08 | 0.06 | 0.17 |  | -0.10 | 0.08 | 0.24 |  | -0.49 | 0.19 | 0.01 |
| Bare ground | 0.01 | 0.06 | 0.89 |  | 0.11 | 0.07 | 0.11 |  | 0.11 | 0.17 | 0.54 |

Table S2a: Model selection results for Red-tailed Hawk (RTHA) and Northern Harrier (NOHA) habitat selection Packsaddle Wildlife Management Areas in Oklahoma, USA, 2013 – 2015. For each species, the top 10 models are presented. Included for each model are number of parameters (K), Akaike’s Information Criterion values corrected for small sample sizes (AICc), delta AICc (ΔAICc), and Akaike weight (Ѡi).

| Models | K | AIC | delta | weight |
| --- | --- | --- | --- | --- |
| *RTHA* |  |  |  |  |
| upland shrub+riperian shrub | 3 | 724.11 | 0 | 0.31 |
| upland shrub+riparian wood | 3 | 724.83 | 0.72 | 0.22 |
| upland shrub+upland wood | 3 | 725 | 0.89 | 0.20 |
| upland shrub+grass | 3 | 725.17 | 1.06 | 0.18 |
| grass | 2 | 728.49 | 4.37 | 0.03 |
| grass+upland shrub+upland wood | 4 | 728.67 | 4.55 | 0.03 |
| riparian wood | 2 | 730.31 | 6.19 | 0.01 |
| pasture | 2 | 730.6 | 6.48 | 0.01 |
| pasture+riparian wood | 3 | 734.52 | 10.41 | 0 |
| riparian wood+grass | 3 | 736.49 | 12.38 | 0 |
| *NOHA* |  |  |  |  |
| grass | 2 | 476.57 | 0 | 0.65 |
| grass+upland shrub | 3 | 478.41 | 1.84 | 0.16 |
| grass+upland wood | 3 | 480.49 | 3.92 | 0.09 |
| grass+bare ground | 3 | 480.66 | 4.09 | 0.08 |
| grass+riparian shrub+riparian wood | 4 | 486.36 | 9.8 | 0 |
| grass+bare ground+upland shrub | 4 | 486.4 | 9.83 | 0 |
| grass+bare ground+riparian shrub | 4 | 486.43 | 9.86 | 0 |
| grass+bare ground+riparian wood | 4 | 487.42 | 10.85 | 0 |
| grass+bare ground+riparian shrub+pasture | 4 | 487.92 | 11.35 | 0 |
| grass+pasture+riparian wood+upland wood | 5 | 492.87 | 16.3 | 0 |
| bare+riparian wood+riparian shrub | 4 | 494.39 | 17.82 | 0 |
| grass+bare ground+upland shrub+riparian shrub+pasture | 6 | 496 | 19.43 | 0 |

Table S2b. Model-averaged coefficients estimating the predicted habitat selection for Red-tailed Hawk, and Northern Harrier plus predicted habitat vulnerability for Northern Bobwhite (NOBO), at Beaver River Wildlife Management Area in western Oklahoma, United States, 2013–2015.

|  | Red-tailed Hawk | | |  | Northern Harrier | | |  | NOBO mortality | | |
| --- | --- | --- | --- | --- | --- | --- | --- | --- | --- | --- | --- |
| Variables | β | SE | Pr(>\|z\|) |  | β | SE | Pr(>\|z\|) |  | β | SE | Pr(>\|z\|) |
| (Intercept) | -0.37 | 0.05 | >0.01 |  | 0.06 | 0.06 | 0.33 |  | -0.03 | 0.07 | 0.70 |
| Grass cover | -0.20 | 0.05 | >0.01 |  | 0.20 | 0.06 | >0.01 |  | 0.06 | 0.05 | 0.07 |
| Pasture | -0.57 | 0.11 | >0.01 |  | 0.60 | 0.13 | >0.01 |  | -0.71 | 0.16 | >0.01 |
| Bare ground | -0.25 | 0.13 | 0.05 |  | -0.05 | 0.06 | 0.42 |  | -0.16 | 0.15 | 0.28 |
| Riparian wood | 0.08 | 0.04 | 0.09 |  | -0.08 | 0.06 | 0.19 |  | -0.15 | 0.16 | 0.34 |
| Upland shrub | -0.05 | 0.04 | 0.28 |  | 0.09 | 0.06 | 0.13 |  | -0.29 | 0.16 | 0.07 |
| Riparian shrub | 0.02 | 0.04 | 0.67 |  | -0.08 | 0.06 | 0.17 |  | -0.55 | 0.17 | >0.01 |

Table S3: Model selection results for Red-tailed Hawk (RTHA) and Northern Harrier (NOHA) habitat selection Beaver River Wildlife Management Areas in Oklahoma, USA, 2013 – 2015. For each species, the top 7 models are presented. Included for each model are number of parameters (K), Akaike’s Information Criterion values corrected for small sample sizes (AICc), delta AICc (ΔAICc), and Akaike weight (Ѡi).

| RTHA | df | AIC | delta | weight |
| --- | --- | --- | --- | --- |
| riparian forest+grass+bare ground | 4 | 1083.04 | 0 | 0.45 |
| riparian forest+grass | 3 | 1083.26 | 0.23 | 0.40 |
| riparian forest+grass+pasture+bare ground | 5 | 1086.75 | 3.71 | 0.07 |
| riparian forest+grass+upland shrub | 4 | 1088.67 | 5.63 | 0.03 |
| riparian forest+grass+upland shrub+bare ground | 5 | 1088.73 | 5.69 | 0.03 |
| riparian forest+grass+riparian shrub | 4 | 1089.84 | 6.8 | 0.01 |
| riparian forest+grass+upland shrub+pasture+bare ground | 6 | 1092.68 | 9.64 | 0 |
| NOHA |  |  |  |  |
| pasture+grass+upland shrub+riparian forest | 5 | 655.89 | 0 | 0.70 |
| pasture+grass | 3 | 659.84 | 3.95 | 0.10 |
| pasture+grass+riparian forest | 4 | 660.15 | 4.26 | 0.08 |
| pasture+grass+bare ground | 4 | 661.3 | 5.41 | 0.05 |
| pasture | 2 | 662.11 | 6.22 | 0.03 |
| pasture+grass+riparian shrub+upland shrub | 5 | 663.93 | 8.03 | 0.01 |
| pasture+grass+upland shrub+riparian wood | 5 | 664.16 | 8.27 | 0.01 |

**Supplementary materials: Figures**

Figure S1: The predicted relative probability of Northern Bobwhite vulnerability (a), Northern Harrier (b), and Red-tailed Hawk (c) habitat selection predicted by resource selection function at Packsaddle Wildlife Management Area in western Oklahoma, USA, 2013–2015.

Fig. S2: The predicted relative probability of Northern Bobwhite vulnerability (a), Northern Harrier (b), and Red-tailed Hawk (c) habitat selection predicted by resource selection function at Beaver River Wildlife Management Area in western Oklahoma, USA, 2013–2015.
